# Mr. Dunning, on Vaccination

**Published:** 1805-05-01

**Authors:** Richard Dunning

**Affiliations:** Plymouth Dock


### To the Editors of the Medical and Physical Journal.

Gentlemen,

On the 1st of this month I vaccinated with the same fluid the two children of a Mr. S. who lives at No. 78, in Pembroke-street, in this town; the eldest is three years old, the youngest eight months: the former has a fair complexion and light hair, and has always been healthy. The vaccinated parts on both arms of this child having taken on a pustulous character, and terminated in ugly yellowish waxy looking scabs, surrounded with an angry fretful redness, I was led very minutely to examine the whole surface of this child's skin. I discovered eight or ten large, foul, nasty blotches, two or three of which were nearly as broad as the nail of my fore finger, and certainly more resembled those we now and then meet with in cases of inveterate or ill cured psora than any other I can refer them to. The scabs of some had fallen off, and the surfaces of these were now scaly. Under one axilla, an excoriated and diffused eruption was much more like the usual appearance of recent scabies on that part. On further inquiry, I found that this child had been more or less subject to blotches of this description from its birth. It may not, perhaps, be out of place to mention, that the father of this child suffered formerly, and not long since, much from the scrophula, under the observation of a very respectable surgeon in this town. The mother is free of every cutaneous disease, and apparently one of the healthiest women in the world. The vesicle on the youngest child's arm (this child has no cutaneous affection) has been correctly expressed, and is now, the thirteenth day, going on to the characteristic black, horny scab.

The preceding case strikingly accords with Dr. Jenner's assertion, that some cutaneous affections now and then suspend or interrupt the regular impression of the vaccine principle. Doctors Remmett and May, Messrs. Little, Dunsterville, Smith, Penkivil, and Bone, did me the favour to visit this child. These gentlemen have, from this case, expressed themselves perfectly convinced of the truth and accuracy of Dr. Jenner's observation. This is not a case of *that incomplete vaccination which I have lately elsewhere adverted*

*adverted to*:—No; in this case there scarcely seems to have been any thing of vaccination; yet would this, from the extent of local affection, &c. in an early period of the practice, have imposed on some. How strongly this case teaches us to see repeatedly, at different periods, the subjects of vaccination. Having, I sincerely hope, done for ever with the small-pox, I am precluded from making an experiment with it on this subject. I have, no doubt, however, that I should equally fail in any attempts to produce a genuine variola on this child during the continuance of this affection on its skin, as I have in my endeavours with the vaccine principle to effect a genuine vaccina. But this analogous inference, as it is open to cavil, will be violently borne down by those persons who *take every thing but give nothing*; by those who, although the Ars Medica, in a general point of view, is confessedly the Ars Conjecturalis, attempt to strangle in birth every conjecture which is forcibly, strikingly, and rationally favourable to the interests of vaccination, and at once quash it as inadmissable in the discussion; at the same time they are insisting on, with every prolixity of detail, a string of greater rhapsodies and more extravagant flights, than were ever till now brought forward on this or any other subject:—in other words, by those who strain at a gnat and swallow a camel. Under such auspices, neither will the liberal science of medicine, nor any other, be expected to make many advances; indeed, his imperantibus, even another Galeleo might tremble for his fate. We shall not in the West Country, however, be soon laughed or frightened out of the little sense we have been blessed with. If this discussion must assume a character of warfare, let the contest be purely English; let it be manly, open, and generous; let us have nothing of assassination in it. I purpose, bye and bye, to give the subject of this case the hydrargyrus cum sulphure; to anoint him with the sulphur ointment; and, if I succeed in removing the disease on his skin, to re-vaccinate him under the observation of the same gentlemen I have mentioned above.

I am, &c.

*Plymouth Dock, Mar.* 13, 1805. RICHARD DUNNING.

